# Annexin A1 in plasma from patients with bronchial asthma: its association with lung function

**DOI:** 10.1186/s12890-017-0557-5

**Published:** 2018-01-04

**Authors:** Sun-Hye Lee, Pureun-Haneul Lee, Byeong-Gon Kim, Hyun-Jeong Seo, Ae-Rin Baek, Jong-Sook Park, June-Hyuk Lee, Sung-Woo Park, Do-Jin Kim, Choon-Sik Park, An-Soo Jang

**Affiliations:** Division of Respiratory and Allergy, Department of Internal Medicine, Soonchunhyang University Bucheon Hospital, 170 Jomaru-ro, Wonmi-gu, Bucheon, Gyeonggi-do 14584 Republic of Korea

**Keywords:** Airway epithelial, Annexin A1, Asthma, Blood, FPR2, Ovalbumin

## Abstract

**Background:**

Annexin-A1 (ANXA1) is a glucocorticoid-induced protein with multiple actions in the regulation of inflammatory cell activation. The anti-inflammatory protein ANXA1 and its N-formyl peptide receptor 2 (FPR2) have protective effects on organ fibrosis. However, the exact role of ANXA1 in asthma remains to be determined. The aim of this study was to identify the role of ANXA1 in bronchial asthma.

**Methods:**

In mice sensitized and challenged with ovalbumin (OVA-OVA mice) and mice sensitized with saline and challenged with air (control mice), we investigated the potential links between ANXA1 levels and bronchial asthma using ELISA, immunoblotting, and immunohistochemical staining. Moreover, we also determined ANXA1 levels in blood from 50 asthmatic patients (stable and exacerbated states).

**Results:**

ANXA1 protein levels in lung tissue and bronchoalveolar lavage fluid were significantly higher in OVA-OVA mice compared with control mice. FPR2 protein levels in lung tissue were significantly higher in OVA-OVA mice compared with control mice. Plasma ANXA1 levels were increased in asthmatic patients compared with healthy controls. Plasma ANXA1 levels were significantly lower in exacerbated patients compared with stable patients with bronchial asthma (*p* < 0.05). The plasma ANXA1 levels in controlled asthmatic patients were correlated with forced expiratory volume in 1 s (FEV_1_) (*r* = − 0.191, *p* = 0.033) and FEV_1_/forced vital capacity (FVC) (*r* = −0.202, *p* = 0.024).

**Conclusion:**

These results suggest that ANXA1 may be a potential marker and therapeutic target for asthma.

## Background

Annexin A1 (ANXA1) is a glucocorticoid-induced protein with multiple functions in the regulation of inflammatory cell activation. ANXA1 is a 37 kDa protein that can bind to cellular membranes in a Ca2 + −dependent manner. The protein has been reported to have anti-phospholipase activity following glucocorticoid induction and possesses a wide range of physiological and pathological functions [1–6]. The biological effects of ANXA1 differ based on intra- versus extracellular localization [7–9]. The extracellular form of ANXA1 stimulates cell motility and cancer cell invasion, mostly via interaction with specific receptors such as the G-protein-coupled formyl peptide receptor (FPR) family [3, 10, 11].

ANXA1 has anti-inflammatory effects by stimulating inflammatory cell programmed cell death and prohibiting eicosanoid synthesis [12, 13]. ANXA1 levels were decreased in smokers or patients with asthma, cystic fibrosis, and rheumatoid arthritis [14–17]. The reduced levels of lipoxin A4 (LXA4) and ANXA1 were reported in wheezy infants [17] and patients with severe asthma [18–20]. In addition, experimental studies have shown that ANXA1 is associated with asthma development [16].

However, the role of ANXA1 in the pathogenesis of asthma is not clear. Using a mouse model, we evaluated ANXA1 expression and levels in the blood of asthmatic patients and evaluated the relationship between ANXA1 levels and clinical profiles in asthma.

## Methods

### Experimental design

Fifty asthmatic patients were recruited and followed for 6.6 ± 3.6 years, and plasma ANXA1 levels were determined during the stable and exacerbated states (Table 1). Experiments involving patients were approved by Soonchunhyang University’s institutional review board (IRB). Eight BALB/c mice were exposed to saline (control) or ovalbumin (OVA). Detailed analyses included evaluation of lung ANXA1 phospho Tyr21 levels and lung histology and FPR2 levels in bronchoalveolar lavage fluid (BALF) and lung tissue. Cells were treated with titanium dioxide nanoparticles (TiO_2_) and dexamethasone (DEX). The animal studies were approved by Soonchunhyang University’s institutional animal care and use committee.

**Table 1:** Clinical characteristics in control subjects and patients with asthma

Characteristic		Control subjects	Asthmatic patients	
			Stable	Exacerbated
No of subjects		25	50	
Sex (male/female)		2/23	20/30	
Age (of initial visit), yr		58.3 ± 6.2	54.9 ± 14.1	
Onset of asthma: age, yr			47.06 ± 17.25	
Asthma duration, yr			6.63 ± 3.60	
Smoking status (NS/ES/CS)		25/0/0	32/12/6	
Cigarettes smoked, pack. yr			9.0 ± 15.7	
Body Mass Index, kg/ m^2^		24.8 ± 2.61	25.4 ± 3.31	
Stable and exacerbate lung function	FEV_1_, % pred.	115.36 ± 16.59	85.43 ± 19.82	62.60 ± 18.14^†^
	FVC, % pred.	96.56 ± 14.51	84.83 ± 16.68	66.51 ± 16.76^†^
	FEV_1_ /FVC	84.24 ± 6.05	74.53 ± 9.72	68.07 ± 11.84^†^
PC20, mg/ml			9.18 ± 10.37	
Total IgE, kU		106.63 ± 188.7	421.5 ± 699.19^*^	
Atopy		1 (4%)	21 (42%)^*^	
Attack average/yr			3.38 ± 3.24	
Duration of exacerbation during follow up			6.63 ± 3.61	
Blood WBC/uL		5587.2 ± 1268.0	7768.8 ± 3448.9^*^	9873.0 ± 4917.1
Blood eosinophil, %		2.73 ± 2.26	5.09 ± 4.97^*^	3.83 ± 5.42
Blood neutrophil, %		56.25 ± 9.8	54.7 ± 12.9	64.6 ± 19.3^†^

Data expressed as mean ± SD. *BMI* body mass index, *ES* ex-smoker, *FEV*_*1*_ forced expiratory volume in one second, *FVC* forced vital capacity, *NS* non-smoker, PC20 methacholine; the concentration of methacholine required to decrease the FEV_1_ by 20%, SM; smoker. ^*^p < 0.01 compared with control subjects, ^†^*p* < 0.05 compared with stable asthmatics

### Subjects

All patients were recruited from Soonchunhyang University, Bucheon Hospital. Asthma diagnoses were based on the Global Initiative for Asthma (GINA) guidelines [21]. This study used the same sample examined by Moon et al. [22] although several different measures and analyses are presented. The biospecimens and data used in this study were provided by the biobank of Soonchunhyang University Bucheon Hospital, a member of the Korean Biobank Network.

All subjects had a clinical diagnosis of asthma supported by one or more of the following criteria: 1) variation in the maximum diurnal peak expiratory flow >20% over the course of 14 days, 2) an increase in forced expiratory volume in 1 s (FEV_1_) of >15% after inhalation of 200–400 μg albuterol, or 3) a 20% reduction in FEV_1_ in response to a stimulating concentration of inhaled methacholine (PC20 methacholine) < 10 mg/ml. All subjects underwent standardized assessments, which included analyses of induced sputum specimens, complete differential blood cell counts, immunoglobulin E (IgE) measurements, chest posteroanterior radiography, allergy skin prick tests, and spirometry. All data were collected at the time of diagnosis, before administration of the asthma medication.

Among asthmatic patients from the hospital, asthmatics matched to normal controls in terms of age, sex, and BMI were selected for the study. Normal control subjects were recruited from among the spouses of the patients or members of the general population who answered negatively to a screening questionnaire regarding respiratory symptoms and other allergic diseases, had FEV_1_ values >80% predicted, PC20 methacholine level > 10 mg/ml, and normal findings on simple chest radiographs. Of the subjects who completed a follow-up period of at least 2 years, 50 were diagnosed using the GINA guidelines [21].

### Asthma exacerbation

Asthma exacerbation was analyzed in subjects who had completed regular follow-up for at least 2 years. Asthma exacerbation was defined by the GINA guidelines as episodes of progressively increasing shortness of breath, cough, wheezing, chest tightness, or some combination of these symptoms, accompanied by decreased expiratory airflow and use of systemic corticosteroids (tablets, suspensions, or injections) or an increase in dose from the stable maintenance dose for at least 3 days and a hospitalization or emergency department visit due to asthma, requiring systemic corticosteroids.

### Elisa

ANXA1 levels in the blood of asthmatic patients was measured by Enzyme-linked immunosorbent assay (ELISA; R&D System, Minneapolis, MN, USA). To compare results from different plates, the optical densities (ODs) of the test samples were adjusted relative to the positive and negative control samples supplied in each kit. The mean OD of duplicate wells was calculated. The index value of each test sample was defined by the following formula: index = (OD of test sample – OD of negative control)/(OD of positive control – OD of negative control) × 100. Low detection limits were set at 0.119 ng/mL for ANXA1 according to the manufacturer’s recommendation.

### Animals

Female 6-week-old BALB/c mice (6 weeks of age, weighing 20–24 g) were purchased from Charles River Korea (Orient Bio Inc., Seongnam, Korea). All mice were sensitized by intraperitoneal injection on days 0 and 14 with 50 μg grade V chicken egg OVA (Sigma-Aldrich, St Louis, MO, USA) emulsified with 10 mg hydroxyl aluminum in 100 μl Dulbecco’s phosphate buffered saline (D-PBS). On days 21–23, all mice received intranasal challenges with 150 μg grade III OVA (Sigma-Aldrich) in 50 μl D-PBS. Control mice were sensitized and challenged with saline. On day 24, airway hyper responsiveness (AHR) was measured, BALF was collected, and lung tissue was processed for protein, RNA, and hematoxylin and eosin (H&E) staining and immunohistochemistry (IHC).

### AHR, BALF, and morphology analysis

Mice were anesthetized with 2.5 mg/kg tiletamine and xylazine (Zoletil and lumpum; Bayer Korea Co, Seoul, Korea), and AHR was assessed following challenges with 0, 5, 20, or 100 mg/ml methacholine (Sigma-Aldrich). Measurements of airway hyperresponsiveness were conducted using an animal pulmonary instrument (OCP-3000) 1 min after each dose with 3 min between doses. The following day, BALF was obtained, centrifuged, and the supernatant stored (−20 °C). The cell pellet was resuspended for cell counting, and cytospin slides were prepared for stained with modified Diff-Quick stain. Differential cell counting was performed on at least 500 cells in each slide using standard morphological criteria under a light microscope. A portion of the lung was fixed in 4% phosphate-buffered paraformaldehyde, embedded in paraffin, sectioned (4 μm), and stained (H&E and IHC staining).

### Immunohistochemistry

Mouse lung sections were deparaffinized and rehydrated in an ethanol gradient series. The sections were incubated with 1.4% hydrogen peroxide in methanol for 30 min to block endogenous peroxidases, with 1.5% horse serum to block non-specific binding, and then with the anti-rabbit ANXA1 primary antibody (1200, Thermo Fisher, Rockford, IL, USA). The next day, sections were treated with the ABC kit (Vector Laboratories, Burlingame, CA, USA). The color reaction was developed by staining with liquid DAB+ substrate (Golden Bridge International Inc., Mukilteo, WA, USA). After immunohistochemical staining, the slides were counterstained with Harris’s hematoxylin for 1 min. Images were analyzed with the ImageJ program (National Institutes of Health, Bethesda, Md).

### Western blot

The extracted lung tissues were homogenized in a protein lysis solution containing 50 mM Tris-HCl (pH 7.4), 50 mM NaCl, 0.1% SDS, 1% Triton X-100, 0.5 mM EDTA, and 100 mM PMSF in distilled water. After centrifuged at 14,000 rpm for 30 min at 4 °C, the soluble materials were collected. Mouse lung lysates and bronchoalveolar lavage fluid (BALF) proteins were separated by SDS-PAGE and transferred to PVDF membranes. The membranes were blocked in TBS with 5% skim milk and 0.1% Tween 20 for 1 h at room temperature before incubating with rabbit anti-ANXA1 (1:1000, Thermo Fisher) or rabbit anti-FPRL1/FPR2 (Novus Biologicals, Littleton, USA) or rabbit anti-ANXA1 (phospho Tyr21) (1:500, Genetex, San Antonio, TX, USA) (overnight, 4 °C). The membranes were then incubated for 1 h at room temperature with an HRP-conjugated secondary antibody (1:5000, Santa Cruz Biotechnology, Dallas, USA). Detection was performed using an enhanced chemiluminescence (ECL) plus Western Blot Detection System (ATTO, Tokyo, Japan) on X-ray film. The relative protein levels were determined by quantitative densitometry and were normalized to anti-β-actin monoclonal antibody (1:5000, Sigma-Aldrich) levels.

### Cell culture

Normal human bronchial primary epithelial cells (NHBE) were purchased from Lonza (Lonza, Basel, Switzerland, cat#. CC-2540). NHBE cells were plated at 3000 cells/cm^2^ in culture 75 cm^2^ flasks in bronchial epithelial cell growth medium supplemented with the BEGM BulletKit™ (Bronchial Epithelial Cell Growth Medium) (Lonza, cat#: CC-3170) and cultured at 37 °C in a 5% CO_2_ incubator. The medium was changed every 48 h, and the cells were grown to 80–90% confluence for 5 to 6 days. NHBE cells (second-passage) with density 1.5 × 10^6^ cells/ml were seeded on in 6-well plate with BEGM medium. The 24 h prior to the experiment, the medium was changed to BEBM basal medium. The cells were exposed to 100 μM TiO_2_ and 10 μM DEX (Sigma-Aldrich, Melbourne, VIC, Australia). Additionally, control cell lines are not exposed to DEX and TiO_2_. Cells were treated with different doses time (4, 8, 24 h).

### Statistical analysis

Receiver operating characteristic (ROC) curve, the area under the curve (AUC) was calculated using significant predictors (as determined via multivariate regression) to derive best suitable cut-off values and to assess model discrimination and predictive accuracy. Determination of optimal cutoff points were conducted with SPSS statistical software package (ver. 20.0; SPSS Inc.; Chicago, IL, USA).

The data were double-entered into the SPSS statistical software package (20).

Data with a normal distribution were described by means and standard deviations and compared using a two-sample *t*-test; data with non-parametric distributions were described by the median and interquartile ranges (IQR) and compared using Mann-Whitney U-test (intergroup analysis) or Pearson’s χ^2^ test for normally distributed, skewed, and categorical data, respectively. Differences between the patient populations were analyzed by χ^2^ test with Fisher’s exact test when low expected cell counts were encountered. Different correlation coefficients were calculated the Spearman’s correlation analysis. *p* < 0.05 was deemed to indicate statistical significance.

## Results

### ANXA1 plasma levels in asthmatic patients

**Fig. 1:**
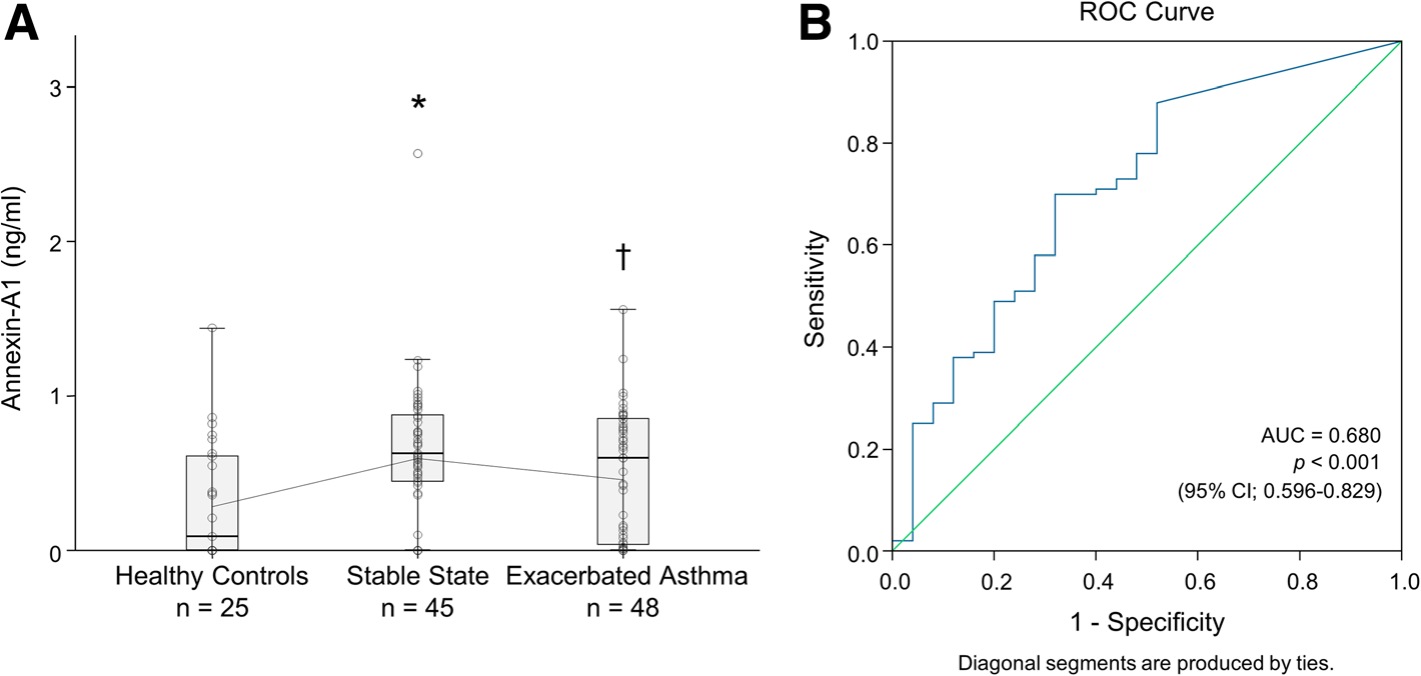
Plasma Annexin-A1(ANXA1) level in healthy control, stable state and exacerbated state of asthmatic patients. **a** Annexin-A1 levels were increased in stable state (*n* = 45) as compared with healthy controls (*n* = 25). Data are box plots with median value and minimal and maximal distribution. *p < 0.01 compared to healthy control subjects. †*p* < 0.05 compared to stable state of asthmatic patients. **b** ROC curves for Annexin-A1 ELISA in human plasma. Comparison between healthy controls (*n* = 25) and asthmatic patients (*n* = 93) was compared (AUC = 0.680, CI 0.596 to 0.829, *p* < 0.001). Diagonal line represents hypothetical curve corresponding to a test with no discriminatory power. AUC, area under the curve; ROC, Receiver operating characteristic

Plasma ANXA1 levels in asthmatic patients (0.570 ± 0.043 ng/ml) were higher than those in healthy control subjects (0.312 ± 0.078 ng/ml; Interquartile range, IQR: 0.0–0.611), (*p* < 0.01) (Fig. 1a). Plasma ANXA1 levels were significantly lower in exacerbated patients (0.497 ± 0.058 ng/ml; IQR: 0.038–0.850) compared with stable patients (0.645 ± 0.061 ng/ml; IQR: 0.447–0.871), (*p* < 0.05) (Fig. 1a). Therefore, this study calculated the best cut-off values of the Annexin-A1 ELISA in patients. By receiver operating characteristic curve (ROC), the best cut-off value of Annexin-A1 ELISA was 0.5023 ng/mL in patients (sensitivity and specificity were 68% and 99.68%, resp.), and the area under curve (AUC) was 0.680 (95% CI; 0.596–0.829), (*p* < 0.001) (Fig. 1b).

### Relationship between ANXA1 levels and clinical variables

**Fig. 2:**
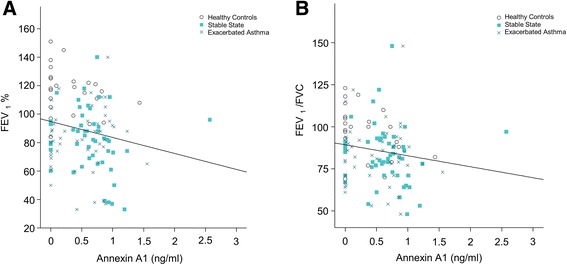
Relationship between plasma AXNA1 level and lung function. Scatter plots of measured values for (**a**) FEV_1_% (spearman, *r* = −0.191, *p* = 0.033) (**b**) FEV_1_/FVC (spearman, *r* = −0.202, *p* = 0.024) against Annexin-A1 levels. Healthy controls (n = 25), stable sate (n = 45), exacerbated asthma (*n* = 48). FEV_1_, forced expiratory volume in 1 s; FVC, forced vital capacity

The population characteristics are shown in Table 1. Plasma ANXA1 levels in controlled asthmatic patients were correlated with forced expiratory volume in 1 s (FEV_1_%) pred. (*r*    = −0.191, *p* = 0.033) (Fig. 2a) and FEV_1_/forced vital capacity (FVC), (*r* = −0.202, *p* = 0.024) (Fig. 2b). Smoking asthmatics tended to have lower levels of ANXA1 compared with non-smoking asthmatics (4.14 ± 0.31 vs. 4.56 ± 0.99 ng/ml; *p* > 0.05). There was no relationship between ANXA1 and age, smoking amount, body mass index, PC20, white blood cell count, or total IgE levels.

### Airway hyperresponsiveness and differential cell counts in mouse asthma model

**Fig. 3:**
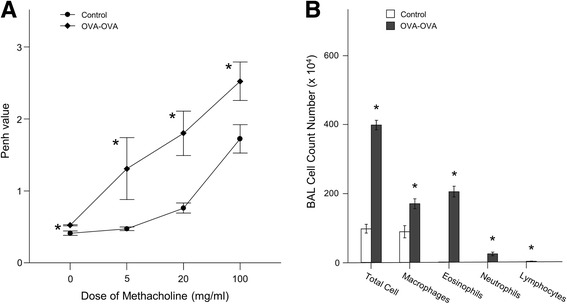
AHR and Differential cell counts in bronchoalveolar lavage (BAL) fluid. **a** AHR developed in the OVA-induced asthma in allergic BALB/c mice group. AHR in the mice treated with increasing concentrations of methacholine (0–100 mg/ml) was measured 24 h following the final OVA challenge (*n* = 8). (*p < 0.05, vs. saline control). AHR, Airway hyperresponsiveness; OVA, ovalbumin. **b** Inflammatory cell counts in BAL fluid obtained from sensitized mice final OVA challenge. Dose-dependently increased OVA-induced inflammatory cell counts in BAL fluid from sensitized mice 24 h after the last OVA aerosol challenge. Cells from BALF were counted, spun onto glass slides and stained with Diff-Quik before microscopy (n = 8). *p < 0.05 compared to control subjects

Airway hyperresponsiveness (AHR) was increased in the OVA sensitized and OVA challenged mice (OVA-OVA mice group) compared with saline sensitized and air challenged mice (Control mice group) (Fig. 3a). Inflammatory cells, including eosinophils, macrophages, lymphocytes and neutrophils were increased in the BAL fluid in OVA-OVA mice compared with control mice group (Fig. 3b). The results demonstrated that induced the infiltration of inflammatory cells into the BAL fluid of the OVA-OVA challenged mice.

### ANXA1 in a mouse model of asthma and NHBE cells

**Fig. 4:**
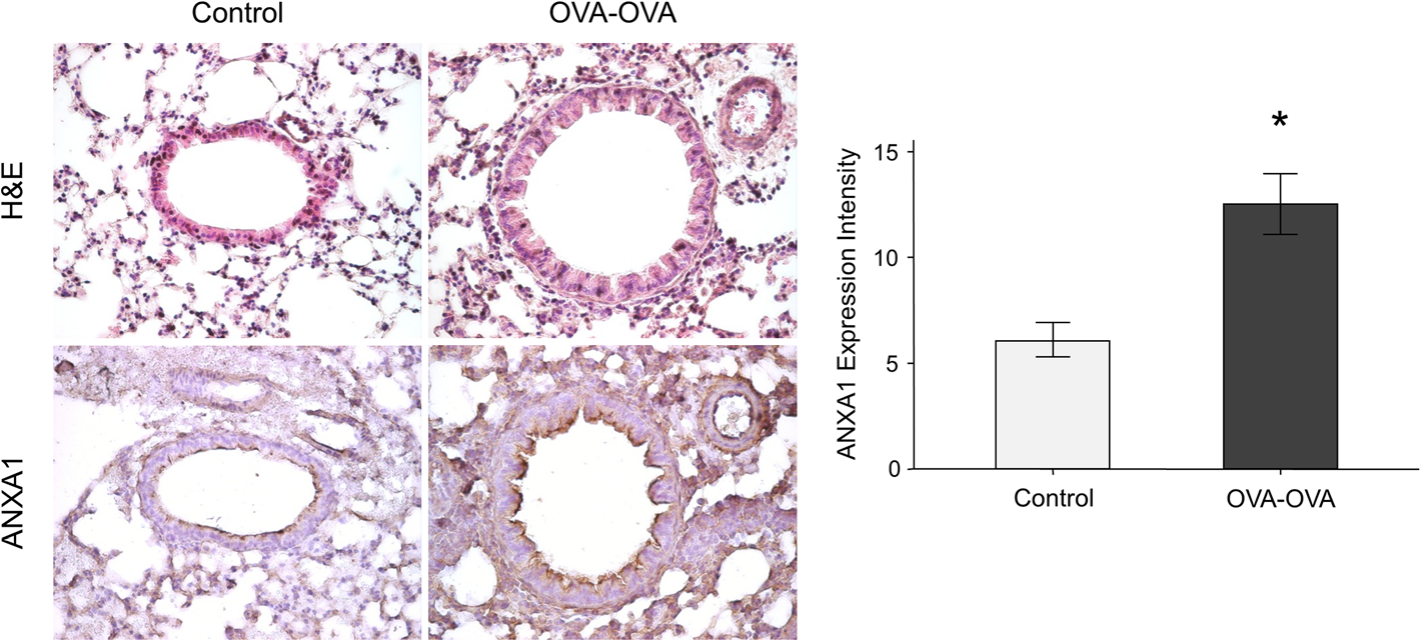
Hematoxylin and eosin (H&E) staining and immunohistochemical staining (IHC) of mouse lung paraffin sections. Lung ANXA1 expression in the mouse lung following ovalbumin (OVA-OVA) sensitization and challenge in immunohistochemical stain with anti-ANXA1 antibody. Increased ANXA1 expression was noted in mononuclear inflammatory cells and endothelial cells, and epithelial cells in OVA-sensitized/challenged mice. *p < 0.05 compared to control subjects

**Fig. 5:**
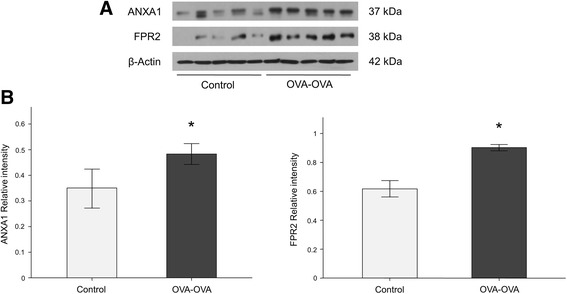
Lung ANXA1 and N-formyl peptide receptor 2 (FPR2) protein levels increase in ovalbumin sensitized and challenged (OVA-OVA) mice. **a** Protein level of ANXA1 and FPR2 as determined by western blot increase in the lung of OVA-OVA mice. **b** Densitometry was determined with 3 immunoblots and normalized to β-actin. The experiment was repeated three times and densitometry was determined with normalized to β-actin. *p < 0.05 compared to control subjects

**Fig. 6:**
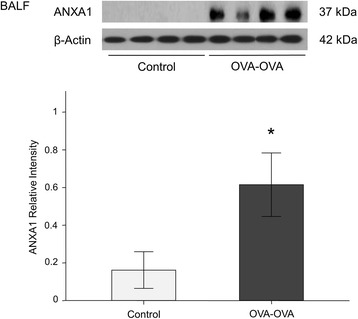
Bronchoalveolar lavage (BAL) ANXA1 protein levels increased in ovalbumin sensitized and challenged (OVA-OVA) mice. Protein level of ANXA1 as determined by western blot increase in Bronchoalveolar lavage fluid (BALF) of OVA-OVA mice. The experiment was repeated three times and densitometry was determined with normalized to β-actin. *p < 0.05 compared to control subjects

**Fig. 7:**
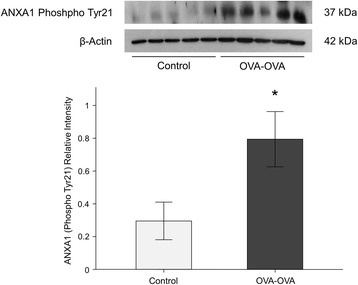
Lung phosphorylated ANXA1 protein levels increased in ovalbumin sensitized and challenged (OVA-OVA) mice. Phosphorylated protein level of ANXA1 as determined by western blot increase in the lung tissue of OVA-OVA mice. The experiment was repeated three times and densitometry was determined with normalized to β-actin. *p < 0.05 compared to control subjects

**Fig. 8:**
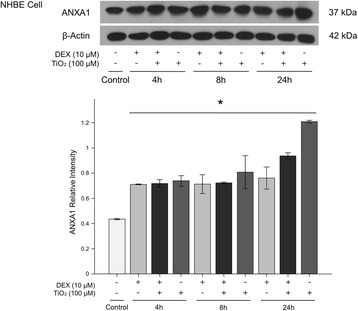
ANXA1 protein levels increased in dexamethasone (DEX) and titanium dioxide nanoparticles (TiO_2_) particles treated NHBE cells at 4, 8, 24 h. Protein level of ANXA1 as determined by western blot increase in extracts of NHBE cells. The experiment was repeated three times and densitometry was determined with normalized to β-actin. *p < 0.01 vs. control

ANXA1 protein levels were significantly increased in lung tissue (Fig. 4) and bronchoalveolar lavage fluid (BALF) (Fig. 5) of mice sensitized and challenged with OVA (OVA-OVA mice) compared with mice sensitized with saline and challenged with air (control mice). FPR2 (Fig. 6) protein levels were significantly increased in the lung tissue of OVA-OVA mice compared with control mice. ANXA1-phospho Tyr21 protein levels were significantly increased in the lung tissue (Fig. 7) of OVA-OVA mice compared with control mice. ANXA1 protein levels were increased (Fig. 8) in dexamethasone (DEX) and titanium dioxide (TiO_2_) treated NHBE cells at 4, 8, 24 h (*p* < 0.05, controls vs. DEX or DEX + TiO_2_ or TiO_2_).

## Discussion

In this study, plasma ANXA1 levels were increased in patients with asthma but decreased in patients with exacerbated asthma. ANXA1 levels were also correlated with lung function, suggesting that ANXA1 may be a potential marker for asthma.

Annexins are soluble proteins that bind to cell membranes containing negatively charged phospholipids, principally phosphatidylserine, in a Ca2 + −dependent manner. The contribution of anti-inflammatory protein ANXA1 and its receptor FPR2 to the regulation of inflammatory responses in human normal lung fibroblasts has been reported previously [23–25]. ANXA1 and FPR2 have protective effects in organ fibrosis [26].

ANXA1, an abundant intracellular protein expressed in many cell types, has known to be induced by glucocorticoids (GCs) and to inhibit phospholipase activity [24–29]. Recombinant ANXA1 or ANXA1-derived N-terminal peptides has similar actions like the anti-inflammatory action of glucocorticoids such as inhibition of inflammatory cells, and suppressing inflammatory mediators [26, 30].

In this study, total and phosphorylated ANXA1 levels were increased in a mouse model of asthma and in inflammatory, endothelial, and epithelial cells. In addition, receptor FPR2 expression was increased in OVA-challenged mice, suggesting that ANXA1 may be involved in asthma pathogenesis.

The ANXA1 receptor FPR2 as a specific G-protein-coupled receptor binds to LXA4 (an anti-inflammatory lipid) and serum amyloid protein, which mediate ligand-specific effects [30, 31]. FPR2 has expressed in human lung fibroblasts, and induced by GCs in human myeloid cells [32, 33].

ANXA1 levels in bronchoalveolar lavage fluids were higher in smokers [14] and patients with cystic fibrosis [15]. A form of ANXA1 with a molecular weight of 33 kDa is released rather than the 37 kDa ANXA1, suggesting that ANXA1 be degraded in smokers and patients with cystic fibrosis [16, 17]. Ng et al. [16] reported that an ANXA-1-deficient mice exhibit spontaneous airway hyperresponsiveness and exacerbated allergen-specific antibody responses in a mouse model of asthma.

In this study plasma levels of ANXA1 were increased in asthmatic patients compared with healthy control subjects, which is similar to the findings of other studies [17–20], suggesting that ANXA1 has compensatory anti-inflammatory effects in asthma. But increased plasma ANXA1 level in stable asthma is decreased in exacerbated asthmatics, indicating that circulating ANXA1 may be decreased due to ANXA1 increase in target inflammatory site in exacerbated state of asthma. To clarify the effect of steroids and particulate matter on ANXA1, we determined the effect of GCs and particulate matter on ANXA1 expression. Dexamethasone and particulate matter induced ANXA1 protein expression, indicating that particulate matter and GCs can activate ANXA1.

## Conclusions

In conclusion, this study demonstrated higher levels of ANXA1 in asthmatic patients and lower levels in exacerbated asthmatic patients. In addition, there was a significant relationship between ANXA1 and lung function in asthmatic patients, indicating that ANXA1 is potentially involved in the pathogenesis of asthma.

Furthermore, the findings in this study indicated that serum ANXA1 concentration represents a biomarker for asthma, which has potential utility as a diagnostic tool. However, larger numbers of asthmatic patients are required for prospective studies, and further studies are warranted to investigate the potential mechanism of ANXA1 in asthma.

## Abbreviations

AHR: Airway hyperresponsiveness
ANXA1: Annexin A1
AUC: Area under curve
BA: Bronchial asthma
BALF: Bronchoalveolar lavage fluid
DEX: Dexamethasone
ELISA: Enzyme-linked immunosorbent assay
FEV_1_: Forced expiratory volume in the first second
FPR2: N-formyl peptide receptor 2
FVC: Forced vital capacity
IQR: Interquartile range
LXA4: Lipoxin A4
OVA: Ovalbumin
ROC: Receiver operating characteristic curve
TiO_2_: Titanium dioxide

## References

[1] Fatimathas L, Moss SE (2010). Annexins as disease modifiers. Histol Histopathol.

[2] Bizzarro V, Fontanella B, Franceschelli S, Pirozzi M, Christian H, Parente L (2010). Role of annexin A1 in mouse myoblast cell differentiation. J Cell Physiol.

[3] Bizzarro V, Petrella A, Parente L (2012). Annexin A1: novel roles in skeletal muscle biology. J Cell Physiol.

[4] Bizzarro V, Fontanella B, Carratù A, Belvedere R, Marfella R, Parente L (2012). Annexin A1 N-terminal derived peptide Ac2-26 stimulates fibroblast migration in high glucose conditions. PLoS One.

[5] Bizzarro V, Belvedere R, Dal Piaz F, Parente L, Petrella A (2012). Annexin A1 induces skeletal muscle cell migration acting through formyl peptide receptors. PLoS One.

[6] Guo C, Liu S, Sun MZ (2013). Potential role of Anxa1 in cancer. Future Oncol.

[7] Lim LH, Pervaiz S (2007). Annexin 1: the new face of an old molecule. FASEB J.

[8] Monastyrskaya K, Babiychuk EB, Draeger A (2009). The annexins: spatial and temporal coordination of signaling events during cellular stress. Cell Mol Life Sci.

[9] Hayes MJ, Rescher U, Gerke V, Moss SE (2004). Annexin-actin interactions. Traffic.

[10] Belvedere R, Bizzarro V, Popolo A, Dal Piaz F, Vasaturo M, Picardi P (2014). Role of intracellular and extracellular annexin A1 in migration and invasion of human pancreatic carcinoma cells. BMC Cancer.

[11] Ye RD, Boulay F, Wang JM, Dahlgren C, Gerard C, Parmentier M (2009). International Union of Basic and Clinical Pharmacology. LXXIII. Nomenclature for the formyl peptide receptor (FPR) family. Pharmacol Rev.

[12] Ferlazzo V, D'Agostino P, Milano S, Caruso R, Feo S, Cillari E (2003). Anti-inflammatory effects of annexin-1: stimulation of IL-10 release and inhibition of nitric oxide synthesis. Int Immunopharmacol.

[13] Parente L, Solito E (2004). Annexin 1: more than an anti-phospholipase protein. Inflamm Res.

[14] Smith SF, Tetley TD, Guz A, Flower RJ (1990). Detection of lipocortin 1 in human lung lavage fluid: lipocortin degradation as a possible proteolytic mechanism in the control of inflammatory mediators and inflammation. Environ Health Perspect.

[15] Vishwanatha JK, Davis RG, Rubinstein I, Floreani A (1998). Annexin I degradation in bronchoalveolar lavage fluids from healthy smokers: a possible mechanism of inflammation. Clin Cancer Res Clin Cancer Res.

[16] Ng FS, Wong KY, Guan SP, Mustafa FB, Kajiji TS, Bist P (2011). Annexin-1-deficient mice exhibit spontaneous airway hyperresponsiveness and exacerbated allergen-specific antibody responses in a mouse model of asthma. Clin Exp Allergy.

[17] Eke Gungor H, Tahan F, Gokahmetoglu S, Saraymen B (2014). Decreased levels of lipoxin A4 and annexin A1 in wheezy infants. Int Arch Allergy Immunol.

[18] Levy BD, Bonnans C, Silverman ES, Palmer LJ, Marigowda G, Israel E (2005). Diminished lipoxin biosynthesis in severe asthma. Am J Respir Crit Care Med.

[19] Planagumà A, Kazani S, Marigowda G, Haworth O, Mariani TJ, Israel E (2008). Airway lipoxin A4 generation and lipoxin A4 receptor expression are decreased in severe asthma. Am J Respir Crit Care Med.

[20] Vachier I, Bonnans C, Chavis C, Farce M, Godard P, Bousquet J (2005). Severe asthma is associated with a loss of LX4, an endogenous anti-inflammatory compound. J Allergy Clin Immunol.

[21] Global Initiative for Asthma. Global Strategy for Asthma Management and Prevention; Web Link: http://ginasthma.org/2017-gina-report-global-strategy-for-asthma-management-and-prevention/. (Accessed 22 Mar 2017).

[22] Moon KY, Lee PH, Kim BG, Park CS, Leikauf GD, Jang AS. Claudin 5 in a murine model of allergic asthma: Its implication and response to steroid treatment. J Allergy Clin Immunol. 2015; 136(6):1694–6.e1–5 10.1016/j.jaci.2015.08.004 26409663

[23] Bouter A, Carmeille R, Gounou C, Bouvet F, Degrelle SA, Evain-Brion D (2015). Review: Annexin-A5 and cell membrane repair. Placenta.

[24] Blackwell GJ, Carnuccio R (1980). Di RosaM, flower RJ, Parente L, Persico P. A polypeptide causing the anti-phospholipase effect of glucocorticoids. Nature.

[25] Miele L, Cordella-Miele E, Facchiano A, Mukherjee AB (1988). Novel anti-inflammatory peptides from the region of highest similarity between uteroglobin and lipocortin I. Nature.

[26] Jia Y, Morand EF, Song W, Cheng Q, Stewart A, Yang YH (2013). Regulation of lung fibroblast activation by annexin A1. J Cell Physiol.

[27] Perretti M, Christian H, Wheller SK, Aiello I, Mugridge KG, Morris JF (2000). Annexin I is stored within gelatinase granules of human neutrophil and mobilized on the cell surface upon adhesion but not phagocytosis. Cell Biol Int.

[28] Mulla A, Leroux C, Solito E, Buckingham JC (2005). Correlation between the anti-inflammatory protein annexin 1 (lipocortin 1) and serum cortisol in subjects with normal and dysregulated adrenal function. J Clin Endocrinol Metab.

[29] Perretti M, Chiang N, La M, Fierro IM, Marullo S (2002). Getting SJ, et al. endogenous lipid- and peptide-derived anti-inflammatory pathways generated with glucocorticoid and aspirin treatment activate the lipoxin A4 receptor. Nat Med.

[30] Fiore S, Maddox JF, Perez HD, Serhan CN (1994). Identification of a human cDNA encoding a functional high affinity lipoxin A4 receptor. J Exp Med.

[31] Le Y, Gong W, Li B, Dunlop NM, Shen W, Su SB (1999). Utilization of two seven-transmembrane, G protein-coupled receptors, formyl peptide receptor-like 1 and formyl peptide receptor, by the synthetic hexapeptide WKYMVm for human phagocyte activation. J Immunol.

[32] VanCompernolle SE, Clark KL, Rummel KA, Todd SC (2003). Expression and function of formyl peptide receptors on human fibroblast cells. J Immunol.

[33] Sawmynaden P, Perretti M (2006). Glucocorticoid upregulation of the annexin-A1 receptor in leukocytes. Biochem Biophys Res Commun.

